# Notes and News

**Published:** 1906-05-05

**Authors:** 


### NOTES AND NEWS.

In consequence of a decided increase in the number of cases of bubonic plague being reported during the past ten days, especially in the provinces of Minieh and Keneh, it has been decided that the annual feast of the Monled el Nebi shall be celebrated on one night only instead of the usual seven, and that measures shall be taken to prevent any large influx of strangers into the country towns. In the week ending April 15 thirty-five cases occurred, and whilst this is not alarming, it is considered that some restriction of the movements of individuals from one district to another is absolutely necessary in the interests of the public.

The dangers of celluloid are pointed out by a resident of Braintree. His daughter has had nearly all her hair burnt off and her scalp roasted, in consequence of a celluloid comb she was wearing catching fire while she was writing near a lamp. The sufferer experienced great agony, and her father states that, but for the unremitting attention of two medical men and a hospital nurse, she would have lost her eyesight, if not her life. She had no idea, he adds, that she was buying a dangerous article. The incident affords further evidence of the urgent necessity of such articles being stamped as "inflammable," in which case purchasers would have only themselves to blame for taking risks.

The Lord Advocate of Scotland, receiving a deputation the other day of various Scottish authorities urging that legislation should be passed to prohibit the sale of tobacco and cigarettes to children on the lines of Dr. Macnamara's Bill, alluded with sympathy to the objects of that measure, but intimated that no suggestion to make criminals of young people for smoking cigarettes could be approved by the Government. He cordially assented to the principle that there should be a check on the sale of a deleterious drug to children, but he saw difficulties ahead in carrying out legislation, such as children getting their elder brothers to buy cigarettes for them, in which cases the vendors would be outside the law.

